# H-Prune through GSK-3β interaction sustains canonical WNT/β-catenin signaling enhancing cancer progression in NSCLC

**DOI:** 10.18632/oncotarget.2169

**Published:** 2014-07-05

**Authors:** Marianeve Carotenuto, Pasqualino De Antonellis, Lucia Liguori, Giovanna Benvenuto, Daniela Magliulo, Alessandro Alonzi, Cecilia Turino, Carmela Attanasio, Valentina Damiani, Anna Maria Bello, Fabiana Vitiello, Rosa Pasquinelli, Luigi Terracciano, Antonella Federico, Alfredo Fusco, Jamie Freeman, Trevor C. Dale, Charles Decraene, Gennaro Chiappetta, Francovito Piantedosi, Cecilia Calabrese, Massimo Zollo

**Affiliations:** ^1^ Centro di Ingegneria Genetica e Biotecnologie Avanzate (CEINGE), Naples, Italy; ^2^ Dipartimento di Medicina Molecolare e Biotecnologie Mediche, Università ‘Federico II’ di Naples, Italy; ^3^ Stazione Zoologica Anthon Dohrn, Villa Comunale, Naples, Italy; ^4^ Dipartimento di Scienze Cardiotoraciche e Respiratorie, Clinica Seconda Università degli Studi di Napoli, Naples, Italy; ^5^ Dipartimento di Pneumologia e Tisiologia, Day Hospital Pneumologia e Pneumoncologico, AORN Vincenzo Monaldi, Naples, Italy; ^6^ Functional Genomic Unit, National Cancer Institute, Fondazione G. Pascale, Naples, Italy; ^7^ Institute of Pathology, Molecular Pathology Division, University of Basel, Switzerland; ^8^ Dipartimento di Biologia e Patologia Cellulare e Molecolare, Istituto Di Endocrinologia e Oncologia Sperimentale del CNR, Naples, Italy; ^9^ School of Biosciences, Cardiff University, Museum Avenue, Cardiff, Wales, UK; ^10^ Translational Research Dept, Institut Curie, Centre de recherche, Paris, France; ^11^ CNRS, UMR144, Paris, France; ^12^ Azienda Ospedaliera Federico II, DAI Medicina Trasfusional, Naples, Italy

**Keywords:** h-Prune, lung cancer, diagnostic marker, WNT/β-catenin signalling, Gsk-3β, Wnt3a

## Abstract

H-Prune hydrolyzes short-chain polyphosphates (PPase activity) together with an hitherto cAMP-phosphodiesterase (PDE), the latest influencing different human cancers by its overexpression. H-Prune promotes cell migration in cooperation with glycogen synthase kinase-3 (Gsk-3β). Gsk-3β is a negative regulator of canonical WNT/β-catenin signaling. Here, we investigate the role of Gsk-3β/h-Prune complex in the regulation of WNT/β-catenin signaling, demonstrating the h-Prune capability to activate WNT signaling also in a paracrine manner, through Wnt3a secretion. In *vivo* study demonstrates that h-Prune silencing inhibits lung metastasis formation, increasing mouse survival. We assessed h-Prune levels in peripheral blood of lung cancer patients using ELISA assay, showing that h-Prune is an early diagnostic marker for lung cancer. Our study dissects out the mechanism of action of h-Prune in tumorigenic cells and also sheds light on the identification of a new therapeutic target in non-small-cell lung cancer.

## INTRODUCTION

H-Prune belongs to the DHH family of phosphoesterases, and it is a human orthologue of *Drosophila* prune, through impairing the formation of drosopterins (red eye pigments). We have reported that h-Prune cAMP-phosphodiesterase (PDE) activity is also involved in cell motility [1]. Kobayashi et al. showed interactions between h-Prune and Gsk-3β, reporting also that Gsk-3β inhibitors and small-interfering RNAs (siRNAs) for GSK-3 or h-Prune inhibit cell motility. These results suggested that h-Prune and Gsk-β cooperatively act to regulate cell motility [2]. Moreover, the minimal domains critical for interaction between h-Prune and Gsk-3β was recently identified between residues Q356 and S396 of C-terminal h-Prune [3].

H-Prune overexpression correlates with T and N stages in colorectal cancer; in addition, h-Prune expression is an independent predictor of survival of patients with gastric cancer [4]. Also, in an analysis of a large set of breast tumours, the pro-motility effects of h-Prune seen *in vitro* translated to significant association with lymph node status and metastasis formation *in vivo*, thus indicating that h-Prune provides a new marker of advanced stage breast carcinoma [5]. Misregulation of the Wnt signalling pathway is a hallmark of many human cancers, including lung cancer [6, 7]. Gsk-3β plays a leading role within the APC complex in initiating proteasomal degradation of β -catenin by phosphorylating it on key residues [8, 9].

In particular when APC complex or Gsk-3β activity is inhibited, the result is protein stabilization of β-catenin allowing it to translocate to the nucleus, where through the bind with TCF/LEF transcription factors, drives the transcriptional activation of genes involved in cell growth, invasion, the stem cell phenotype, and metastasis [8].

Here we studied h-Prune expression in lung cancer, showing its up-regulation during cancer progression. Then to better understand the molecular events in which h-Prune is involved and to clarify its effects on cell malignancy, we examined the role of h-Prune in the control of cell proliferation, cell cycle, apoptosis and cell invasion in vitro. Additionally, being h-Prune a binding partner for Gsk-3β, we investigated how h-Prune/Gsk-3beta interaction was able to affect Wnt signaling pathway.

We demonstrated h-Prune capability to induce activation of canonical WNT/β-catenin signaling by promoting sequestration of Gsk-3β inside multivesicular bodies (MVBs), which are essential components of the WNT signal-transduction pathway [10], further showing the ability to activate WNT pathway in a paracrine manner, through Wnt3a increased secretion.

Finally, we evaluated the value of h-Prune and Wnt3a as tumour markers investigating their protein levels in peripheral blood of lung cancer patients. Here we show that the mechanism of action by which h-Prune enhances canonical WNT pathway activation, and its correlation to disease progression, provide the basis for the definition of a new diagnostic marker to detect NSCLC.

## RESULTS

### H-Prune protein expression enhances WNT pathway activation

**Figure 1:**
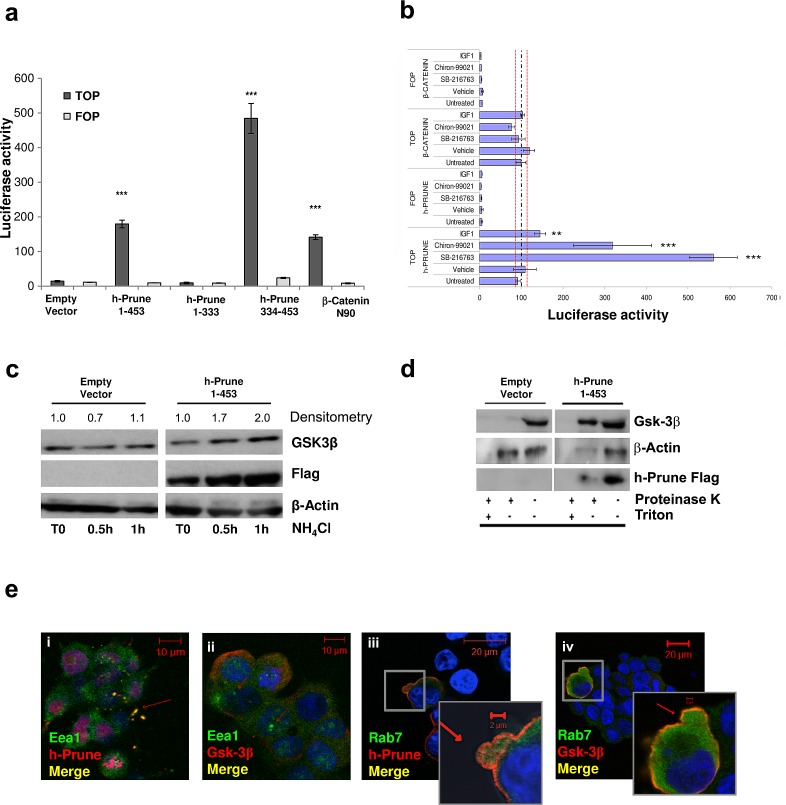
H-Prune expression enhances Wnt pathway activation (a) Full-length h-Prune (aa 1-453) and h-Prune C-terminal region (aa 334-453) stimulation of HEK293 cells induces β-catenin-mediated transcriptional activity. The cells were transfected with the TOP/FOP flash plasmid and the Renilla luciferase plasmid overnight. The data were normalized to Renilla luciferase activity. Full-length h-Prune and h-Prune C-terminal region increased TCF transcriptional activity in HEK293 cells compared to empty vector and h-Prune N-terminal region (1-333). (b) TOP/FOP luciferase assay that shows that h-Prune induces β-catenin–mediated transcriptional activity through a GSK-3β–dependent mechanism in HEK293 cells. The cells were co-transfected with the TOP/FOP luciferase reporter, the renilla luciferase plasmid, and the control or h-Prune vector. A day after infection, the cells were treated overnight with vehicle, SB216763 (10 μM), Chiron99021 (10 μM) and IGF-1 (1 ng/ml per 5h). The data were normalized to Renilla luciferase activity and are expressed as fold-increase over vector-transfected control cells, expressed as means ±SD of three independent experiments, each carried out in triplicate. (c) Total protein prepared from HEK293 cells transfected with control vector or full-length h-Prune, which were exposed to NH_4_Cl (30 mM) for different times, and were subjected to Western blotting with an anti-GSK3-β antibody. α-Tubulin was used as the loading control, and an anti-Flag antibody provided the transfection control. (d) Western blots of protease protection assay in empty-vector-and h-Prune-transfected HEK293 cells. Cells were digitonin-permeabilized, and membranes were isolated and incubated without or with proteinase K in the absence or presence of Triton X-100. Gsk-3β was protected from Proteinase K in h-Prune overexpressing cells, but only in the absence of Triton X-100. (e) Representative images from immunofluorescence analysis of HEK293 cells transfected with plasmid encoding full-length h-Prune and stained for Gsk-3β and the early endosome marker EEA1 (ii), and for h-Prune and EEA1 (i). Scale bars, 10 μm. (iii, iv) Representative images from immunofluorescence analysis of HEK293 cells transfected with the plasmid encoding full-length h-Prune, and stained for Gsk-3β and the late endosome marker Rab7 (iv), and for h-Prune and Rab7 (iii). Scale bars, 20 μm.

H-Prune interacts with Gsk-3β, and together they are involved in regulation of cell migration through the modulation of focal adhesions [2]. *In-vitro* binding studies using recombinant proteins have demonstrated that the carboxy-terminus amino-acid region 333-453 of h-Prune is necessary and sufficient for complex formation with Gsk-3β, with the identification of a minimal region of interaction of the h-Prune protein [2, 3]. Moreover, one of the main functions of Gsk-3β is the regulation of β-catenin turnover in the WNT signaling pathway. For this reason, we asked whether the h-Prune/Gsk-3β interaction can also be identified in lung cancer cell lines, and if this interaction has a role in canonical WNT signaling. First, we performed co-immunoprecipitation assay and observed that endogenous Gsk-3β interacts with h-Prune-Flag in A549 lung cancer cell line (Supplementary Fig. S1a). Next, we examined whether the interaction between h-Prune and Gsk-3β has a functional role for TCF transcriptional activity. To evaluate this, we used TOP-FLASH reporter [11] assays in the HEK293 cell line, in which we observed low levels of the endogenous h-Prune protein (Supplementary Fig. S1c). As shown in Figure 1a, compared to the empty vector transfected cells, there were high levels of endogenous β-catenin transcriptional activity in HEK293 cells transfected with h-Prune full-length (aa 1-453; *P*= 9.4e^-06^) and h-Prune C-terminal region (aa 334-453; *P*= 2e^-05^). To ensure that TOP-FLASH activation was due to h-Prune/Gsk-3β interaction, we also transfected HEK293 cells with plasmid encoded h-Prune N-terminal region (aa 1-333), that does not bind Gsk-3β, as demonstrated by Kobayashi and colleagues [3]. Our results demonstrated that there wasn't TOP-FLASH activation in h-Prune N-terminal transfected cells, thus strengthening our hypothesis. The additional data presented in Supplementary Fig. S1d show that h-Prune can activate the TOP-FLASH in a dose-dependent manner, thus showing that h-Prune is an activator of WNT signaling.

To gain insight into the mechanism accounting for the enhancement of β-catenin-mediated TCF transcriptional activity in HEK293 cells, we also used several known Gsk-3β inhibitors coupled with h-Prune transfection, to evaluate whether or not there was further enhancement of the activation of TCF transcriptional activity. Figure 1b shows the enhancement of transcriptional activity in the h-Prune-transfected cells, and in cells transfected with plasmid encoding h-Prune and also treated with SB-216763, Chiron-99021 and/or insulin growth factor-1 (IGF-1). This was not observed using β-catenin cDNA transfection in these cells. The simplest explanation would be that there is a cooperative effect between h-Prune and the other drugs used here, for the inhibition of β-catenin degradation by Gsk-3β. Moreover, both increased expression levels of active β-catenin and inhibition of its degradation were enhanced by h-Prune overexpression, as additionally confirmed by the Western blotting (Supplementary Fig. S1e and f). Collectively, these data demonstrate that the binding of h-Prune and Gsk-3β is sufficient to mediate the inhibition of β-catenin degradation, thus leading to an enhancement of the free β-catenin levels for its translocation into the nucleus and the activation of WNT signaling.

### H-Prune overexpression leads to increased Gsk-3β sequestration inside multivesicular bodies

Taelman and colleagues demonstrated that Gsk-3β sequestration inside MVBs is a key step in WNT/β-catenin signaling activation [10]. In this study, we explored whether h-Prune can induce Gsk-3β sequestration inside MVBs. To investigate this, we first treated HEK293 cells with NH_4_Cl, a known inhibitor of acidification and organisation of the endosomal compartments [13]. This treatment was carried out 36 h post-transfection with a control empty vector and a plasmid encoding h-Prune, with the cells harvested at different times. As shown in Figure 1c, 0.5 h and 1 h after NH_4_Cl administration there was an increase (with 2-fold increase with respect to Time 0, as shown by densitometric analyses) in Gsk-3β protein levels in the h-Prune-overexpressing cells, as compared to empty-vector-transfected cells, suggesting an increase of Gsk-3β in endosomal compartment in h-Prune-ovexpressing cells. Next, we performed a protease protection assay, the golden standard analysis to determine the localization of a protein in a membrane-bounded compartment. Here, the Gsk-3β and h-Prune proteins were protected from proteinase K digestion after h-Prune transfection in HEK-293 cells, as compared to the empty-vector-transfected cells (Figure 1d). This phenomenon whereby h-Prune provides protection against proteinase K degradation of Gsk-3β was abolished when the membranes were solubilized with Triton X-100. Furthermore, in h-Prune-overexpressing cells, fluorescence microscopy (Figure 1e) showed co-localisation of Gsk-3β and Flag-tagged h-Prune with the early and late endosomes markers (EEA1 and Rab7), thus leading us to speculate upon a physical interaction between h-Prune and GSK-3β, such that both of these proteins move into endosomes during WNT activation.

### H-Prune-conditioned medium induces WNT signaling

**Figure 2:**
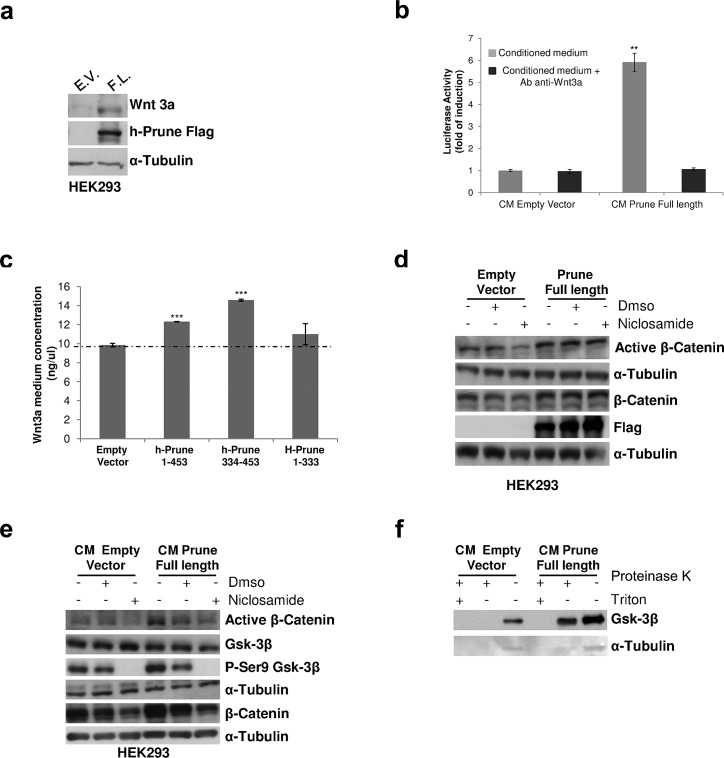
H-Prune–conditioned medium induces Wnt signalling activation (a) Western blotting showing that overexpression of h-Prune leads to increased Wnt3a expression, as compared to empty-vector-transfected cells. The efficiency of transfection was assessed using an anti-Flag antibody. α-Tubulin was used as the loading control. (b) TOP/FOP assay performed on reporter cells stimulated with equal amounts of conditioned medium (CM) from HEK293 cells transfected with either the empty vector plasmid or the plasmid encoding h-Prune. *P = 0.01. Treatment with the anti-Wnt3a antibody reduced activation of TOP/FOP in cells receiving conditioned medium from HEK293 cells transfected with the plasmid encoding h-Prune. This further indicates that Wnt3a released into the medium can activate the Wnt signaling in the neighbouring cells in a paracrine manner. (c) ELISA to determine Wnt3a release in the medium from HEK293 cells transfected with the empty vector plasmid or plasmids encoding full-length h-Prune (aa 1-453), h-Prune C-terminal region (aa 334-453) or h-Prune N-terminal region (aa 1-333). ***P>0.0005. (d) Total protein prepared from HEK293 cells after transfection with the control vector or full-length h-Prune, which were exposed to niclosamide (0.6 μM) for 6 h were subjected to Western blotting with anti-β-catenin and active β-catenin antibodies. α-Tubulin was used as the loading control and anti-Flag antibody provided the transfection control. (e) HEK293 cells were stimulated for 6 h with conditioned media (CM) from HEK293 cells transfected with empty vector and h-Prune vector. Niclosamide or DMSO (as control) were added to the conditioned media. Protein extracts were loaded onto acrylamide gels and subjected to Western blotting with anti-β-catenin, active β-catenin, GSK3-β and pSer9 GSK3-β antibodies. α-Tubulin protein levels were used as the loading control. (f) HEK293 cells were stimulated for 6 h with conditioned media (CM) from HEK293 cells transfected with empty vector and h-Prune vector. The protease protection assay shows Gsk-3β protected from Proteinase K in cells receiving CM from h-Prune overexpressing cells.

The activation of TCF/LEF transcription has been shown to have negative and positive feedback loops on WNT pathway activation [14, 15]. To investigate whether h-Prune overexpression has this signal-inducing activity, we first quantified the expression levels of several WNT proteins, both canonical and non canonical, in HEK293 cells following h-Prune transfection (Supplementary Fig. S2a). The qPCR showed that the overexpression of h-Prune significantly enhanced WNT3a expression to a level 4-fold the control (*P*=0.045; Supplementary Fig. S2a), while there were no significant differences in the mRNA expression of the other analyzed cytokines. Therefore, we evaluated WNT3a protein expression levels, which confirmed the mRNA expression results (Figure 2a). These results led us to investigate whether there was an enhancement of Wnt3a release that could exert paracrine effects on the other adjacent cells. To evaluate these paracrine effects induced by h-Prune overexpression, conditioned medium of h-Prune-transfected cells was added to cells transiently transfected with the TCF/LEF reporter. As shown in Figure 2b, by comparing the TCF/LEF transcriptional reporter activity in HEK-293 cells, we observed that the addition of medium from cells transfected with the plasmid encoding h-Prune led to six-fold induction of WNT activity, whereas no induction was observed by adding medium from empty-vector-transfected cells (*P*= 0.003). Under the same conditions, the addition of Wnt3a neutralizing antibody downregulated reporter activity in HEK293 cells incubated with h-Prune overexpressing cells conditioned medium to levels similar to that observed in cells treated with CM from empty vector-transfected cells. If h-Prune-conditioned medium can lead to the activation of WNT signaling, we reasoned that h-Prune can induce cytokine release that in turn will activate the signals, thus influencing adjacent cells in a paracrine manner. Furthermore, to confirm the release of Wnt3a into the cell medium, we performed an ELISA for Wnt3a. Here, the HEK293 cells were transfected with a plasmid encoding full-length h-Prune (aa 1-453), the h-Prune C-terminal region (aa 334-453 responsible for binding with GSK3-β) or the h-Prune N-terminal (aa 1-333) as control. When compared to transfection with the empty vector, we observed significant enhancement (h-Prune full length *versus* empty vector *P=* 3.7 e^−05^; h-Prune C-terminal region *versus* empty vector *P=* 8.3 e^−05^) of the release of the cytokine Wnt3a into the medium (Fig. 2c; Supplementary Fig. S3a). While, as expected, when compared the transfection with plasmid encoding h-Prune N-terminal to empty vector, no significant enhancement of the release of the cytokine Wnt3a into the medium was observed (*P=* 3.7 e^−05^).

In our previously reported data, we showed enhancement of Wnt3a levels following h-Prune over-expression. Here, to exclude further that nuclear translocation of β-catenin is exclusively due to the positive feedback within the cell by Wnt3a, we used niclosamide, which is known to damage Wnt3a/β-catenin signaling activation by inducing LRP6 protein degradation [16]. First, we evaluated the active β-catenin protein levels upon 0.6 μM niclosamide treatment in both empty vector- and h-Prune-overexpressing HEK293 cells. Western blotting demonstrates that h-Prune can increase the protein levels of active β-catenin even in niclosamide-treated cells, unlike what was seen in the empty-vector-transfected cells (Fig. 2d). Then, we determined whether h-Prune-conditioned medium still activated canonical WNT signaling in these niclosamide-treated cells. Interestingly, addition of conditioned medium from h-Prune-transfected HEK293 cells resulted in increased levels of active β-catenin (Fig. 2e) in the receiving HEK293 cells. Furthermore, addition of niclosamide to the conditioned medium led to a reduction in active β-catenin levels in cells that received the conditioned medium from the empty-vector-transfected and h-Prune-transfected cells.

Several studies demonstrated that Wnt3a induces endocytosis of Gsk-3β into MVBs, thus leading to WNT pathway activation [10, 17]. This result was reproduced in HEK293 cells treated for 6 hours with conditioned medium from empty vector and h-Prune overexpressing cells. The results in figure 2f show that h-Prune conditioned medium is able to induce Gsk-3β uptake into MVBs in the receiving HEK293 cells. While in cells receiving CM from control cells, we didn't observe this phenomenon. Overall, these data strengthen our previous findings, confirming that h-Prune can activate the canonical WNT/β-catenin signaling through Gsk3β interaction and is also capable of inducing the activation of WNT signalling in a paracrine manner through the release of Wnt3a into the medium.

### H-Prune promotes β-catenin nuclear translocation through a GSK-3β–dependent mechanism

In the WNT/β-catenin pathway, the N-terminal phosphorylation of β-catenin by GSK-3β is a key control point for ubiquitin-dependent β-catenin degradation [18]. To gain further insight into the mechanism of β-catenin nuclear translocation mediated by h-Prune, we asked whether h-Prune is necessary for β-catenin degradation in lung cancer cells.

**Figure 3:**
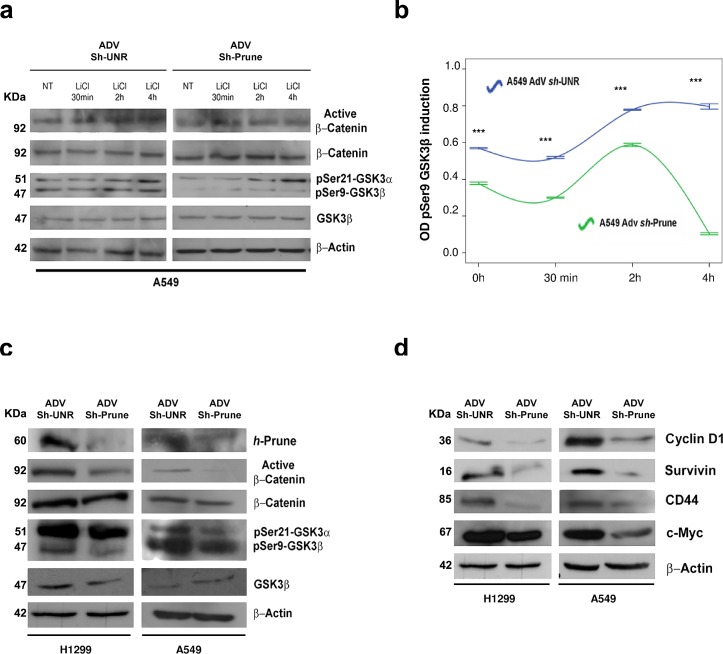
Regulatory mechanism of h-Prune on Gsk-3β (a and b) Effects of LiCl (20 mM) on β-catenin nuclear translocation and phosphorylation status of Gsk-3β in A549 AdV-*Sh*-UNR- and AdV-*Sh*-Prune-infected cells for different times was assayed by Western blotting analysis (a). Densiometric time-course analysis (pSer9-Gsk-3β), as β-actin normalized. Data are means ±standard deviation of 3 experiments, each carried out in triplicate (b). (c) After h-Prune depletion in H1299 and A549 cells, Western blotting detected decreased expression of active β-catenin and pSer9-Gsk-3β. Total β-catenin, total Gsk-3β and β-actin were used as the loading controls. (d) Expression levels of Cyclin D1, Survivin, CD44 and c-Myc decreased with h-Prune silencing. β-Actin was used as the loading control.

If h-Prune impairs GSK-3β activity, we speculated that the negative effects of h-Prune knockdown on β-catenin activation should be ameliorated by the loss of functional activity of GSK-3β. To test this hypothesis, we examined the consequences of the treatment of h-Prune-knockdown A549 cells with LiCl, a specific inhibitor of Gsk-3β (Fig. 3a and b). To achieve this, using LiCl, we evaluated the levels of active β-catenin and phospho-Gsk-3β (on Ser9) in a cellular context without and with h-Prune expression, and at different times. In these assays, LiCl stimulation led to early activation of β-catenin at 30 min of treatment, followed by increased β-catenin protein levels between 2 h and 4 h post-treatment. We thus observed that the protein levels of phospho-Gsk-3β (Ser9) were up-regulated at the same time after induction in cells infected with an unrelated shRNA (sh-UNR). On the other hand, in AdV-*Sh*-Prune-infected cells, where the levels of phospho-Gsk-3β were already decreased, there were no substantial variations in the activation of β-catenin, and nor was there any enhancement of phospho-Gsk-3β (Ser9) protein levels at either 2h and 4h of treatment (Fig. 3a and b). Altogether these data indicate that β-catenin nuclear translocation followed by LiCl-induced canonical WNT signaling activation in lung cancer cells requires the h-Prune inhibitory effects on Gsk-3β activity. Furthermore, the results presented here suggest that h-Prune/Gsk-3β binding results in significant alteration to WNT/β-catenin signaling.

### WNT pathway impairment by h-Prune silencing in lung cancer cell lines

We additionally evaluated the effects of h-Prune silencing on WNT target genes. For this purpose, we used AdV-*Sh*-Prune and an adenovirus containing an unrelated shRNA (AdV-*Sh*-UNR). Both of these were constructed such that they could be followed through internal ribosomal entry site-driven green florescent protein (GFP). H1299 and A549 lung cancer cell lines were then transduced by infection with adenovirus particles, and 24 h later, the efficiency of infection was evaluated through GFP expression (Supplementary Fig. S4b). At 72 h after infection, in AdV-*Sh*-Prune–infected lung cancer cell lines, h-Prune was significantly down-regulated (Fig. 3c), while no inhibition was observed in the AdV-*Sh*-UNR–infected cells. As shown in Figure 3c, the expression of active β-catenin was reduced upon h-Prune down-regulation, while the levels of total β-catenin were unchanged. Consistent with these data, we also observed that the protein levels of cyclin D1, Survivin, CD44, and c-Myc, as targets of canonical WNT signaling, were significantly down-regulated in these AdV-*Sh*-Prune–infected A549 and H1299 cells, with respect to the Adv-*Sh*-UNR–infected cells (Fig. 3d). Furthermore, by silencing the endogenous h-Prune in H1299 and A549 cells, there were reduced mRNA expression levels of Birc5, c-Myc and CD44, supporting the concept that h-Prune regulates these targets via inhibition of transcription (Supplementary Fig. S4c). Altogether, these data demonstrate that while h-Prune overexpression leads to canonical WNT activation, conversely, its loss of function through RNA interference, leads to an impairment of WNT activity in lung cancer cell lines that then inhibits several target proteins downstream of this inhibition.

### h-Prune silencing influences proliferation and soft agar colony formation in lung cancer cells

**Figure 4:**
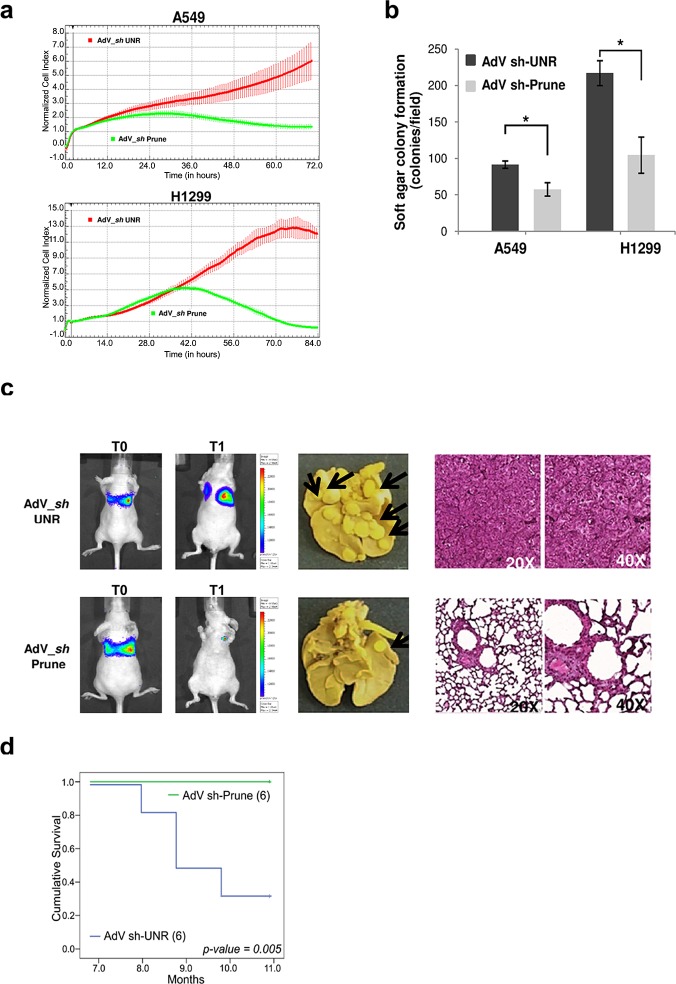
H-Prune silencing effects on proliferation of lung cancer cells (a) Normalized cell index as a measure of proliferation of A549 (upper) and H1299 (lower) cells treated with AdV-*Sh*-UNR and AdV-*Sh*-Prune. Data are means ± SD. (b) A549 and H1299 cells were infected with AdV-*Sh*-UNR and AdV-*Sh*-Prune and plated at 1.5×10^5^ cells/well in six-well plates for the soft agar colony assays. Data are means ±SD (n = 2). H-Prune silencing decreased the soft agar colonies in both lung cancer cells (*P = 0.04 and 0.03, respectively). (c) The AdV-*Sh*-UNR (6 mice) or AdV-*Sh*-PruneA549_Luc cells were injected into SCID mice by tail-vein injection, and lung colonization were bioluminescently imaged at the time of injection (T0) and after 11 months (T1) (left). Representative images of the lungs of A549-Luc AdV-*Sh*-UNR and AdV-*Sh*-Prune–treated mice, macroscopically examined (in the middle) and stained with hematoxylin-eosin after death (A549-Luc AdV-*Sh*-UNR–treated mice) or at the end of the trial (AdV-*Sh*-Prune–treated mice) (right). (d) Kaplan-Meier survival curves for nude mice tail-vein injected with A549_Luc AdV-*Sh*-UNR (6 mice) or AdV-*Sh*-Prune (6 mice).

We then assay dynamics of cell growth and proliferation. Using cell index assays, we confirmed that with both of these lung cancer cell lines, when they were infected with AdV-*Sh*-Prune they showed impaired proliferation, compared to AdV-*Sh*-UNR–infected cells (Fig. 4a). Soft agar colony-formation assays were then performed to determine whether down-regulation of h-Prune reduces colony formation in an anchorage-independent culture system (Fig. 4b). In these assays, the number of colonies of the AdV-*Sh*-UNR–infected *versus* AdV-*Sh*-Prune–infected H1299 cells were significantly reduced: 217 (±16.9) *versus* 104.5 (±24.7), respectively (*P*= 0.03). There was a similar significant reduction in the infected A549 cells: 91.5 (±4.9) *versus* 57.5 (±9.19), respectively (*P*= 0.04; Fig. 4b). Therefore, h-Prune silencing also reduces anchorage-independent cell proliferation of lung cancer cells.

### *In-vivo* lung metastasis assay using xenografts in nude mice

As h-Prune silencing suppressed the invasion and proliferation of NSCLC cells *in vitro*, we sought to investigate whether h-Prune silencing can inhibit metastases formation of lung cancer cells *in-vivo* using a mouse lung metastasis model. To achieve this, we assayed two groups of xenograft mice that were tail-vein injected with A549-Luc cells (A549 cells stabilised for the expression of the luciferase gene); these cells were previously infected with the control AdV-*Sh*-UNR or AdV-*Sh*-Prune. Tumorigenesis was followed using *in-vivo* biolumuniscence imaging (BLI) technology, over 11 months. The mice receiving AdV-*Sh*-Prune-treated A549-Luc cells (6 mice) showed significant reduction of tumour burden (Fig. 4c) compared to the control treated group (AdV-*Sh*-UNR, 6 mice).

In the same experiment, we investigated the survival rates of these mice. The animals were observed daily until their death. At month 11, all of the surviving mice were sacrificed and their lungs were excised and stained with Bouin's solution, with the incidence of tumour nodules examined macroscopically. As shown in Figure 4c, compared to the control A549 lung cancer cells that were treated through AdV-*Sh*-UNR infection, silencing h-Prune expression in these cells resulted in decreased lung metastasis nodules, thus further suggesting that h-Prune is a positive regulator of cancer invasion and metastasis formation *in vivo*. Moreover, the Kaplan-Meier survival curves for these treated mice revealed that the silencing of h-Prune directly and significantly correlates to overall survival (*P*= 0.005) (Fig. 4d). Taken altogether, our data demonstrate that h-Prune is necessary to maintain the level of malignancy in lung cancer, in terms of tumour metastasis formation, invasion and growth.

### h-Prune expression in human NSCLC tissues

To assess the expression levels of h-Prune in patients with NSCLC, we tested a cohort of 45 human tumour samples that had been flash-frozen after biopsy and before RNA isolation. The sample collection was composed of the predominant histotypes of NSCLC. H-Prune expression levels in the tumour tissues were assessed using a custom-made Illumina microarray platform, and these were compared with the levels in the corresponding normal adjacent tissue (Supplemental Table S1). Of the 36 samples analysed, significantly increased h-Prune expression levels were seen in tumour tissues (stage I and stage II; n = 28) compared to the corresponding normal adjacent lung tissues (n = 8; *P*=0.018) (Supplementary Fig. S6a). To additionally validate the microarray gene-expression data, the relative expression of h-Prune was determined by qRT-PCR analysis of 33 samples from the same dataset (as 8 normal tissues and 25 tumour tissues). These were combined with a second dataset of 12 lung tissues (as 7 normal tissues and 5 tumour tissues) for a total of 30 tumour and 15 normal samples. These thus confirmed the previous expression data, showing higher h-Prune expression levels in stage I-II lung cancer tumours (*P*=0.008), compared to the normal lung tissues (Supplementary Fig. S6b). Moreover, after analysis, the overexpression of h-Prune was also observed in additional published lung cancer dataset [19-22], as reported in Supplemental Figure S6c.

### Immunohistochemical analysis of h-Prune in lung cancer

**Figure 5:**
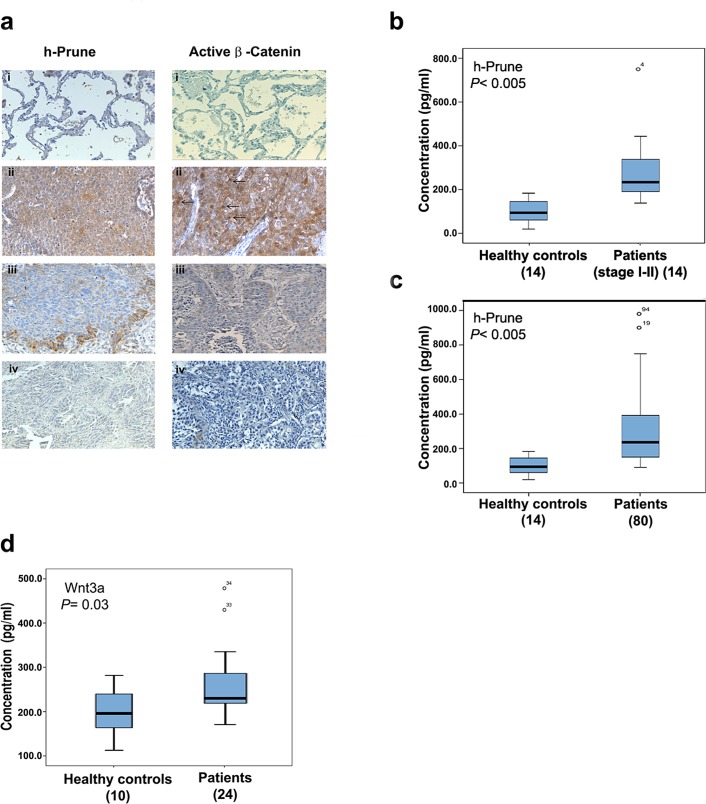
H-Prune is overexpressed in human NSCLC (a) Representative paraffin sections analyzed by immunohistochemistry using a home-made anti–h-Prune rabbit polyclonal antibody. (i) Immunostaining of lung normal tissue (200×). No immunoreactivity was observed. (ii) Immunostaining of a carcinoid tissue (400×). Hyperplastic cells show weak cytoplasmic staining. (iii) Immunostaining of a lung adenocarcinoma (200×). Cytoplasmic staining was observed. (iv) Immunostaining of a squamous cell lung carcinoma (200×). Cytoplasmic positivity was observed in malignant cells. (b) Box plot showing the serum h-Prune levels in 14 healthy controls and 14 patients with stage I-II NSCLC (***P> 0.0005). (c) Box plot showing the serum h-Prune levels in 14 healthy controls and 80 patients (***P> 0.0005). (d) Box plot showing the serum levels of Wnt3a in 10 healthy controls and 24 patients (*P= 0.03).

Immunohistochemical analysis of h-Prune has been reported in several types of cancers [5, 23]. We next sought to determine whether h-Prune overexpression indeed contributes to β-catenin activity in lung cancer tissues. For this purpose, we determined both h-Prune and active β-catenin expression in 33 primary human lung cancer tissues using immunohistochemical staining assays. It has been well documented that the β-catenin accumulated in the cytoplasm and/or the nucleus increases when cells have stabilised β-catenin and, consequently, activated β-catenin/TCF activity. In contrast, when β-catenin is mainly localised at the plasma membrane of cells, it is known that the transactivation activity is low. As shown in Figure 5a, there were low h-Prune and β-catenin protein expression levels in normal adult lung tissues. In contrast, h-Prune expression was detectable in 30 of 30 (100%) lung cancer samples. Of those positive samples, 5 showed limited reactivity (score +1), 4 showed moderate reactivity (score +2), and 21 (70%) showed strong reactivity (score +3). In most cases, the h-Prune staining was also localized to the cytoplasm. In all, 90% of the tissues also showed positive immunohistochemical staining for β-catenin. We also observed no significant variations in the h-Prune expression patterns between the different histological types of lung cancer (Supplementary Table S2). Altogether, these data not only support our previous molecular data, but also further strengthen the clinical biological significance of h-Prune expression in lung cancer patients.

### Serum levels of h-Prune expression in lung cancer patients

To date, most lung cancer patients are diagnosed at an advanced stage of disease, making curable surgery not an option; thus, early diagnosis and effective treatment are key to prolong the survival of lung cancer patients. To evaluate the importance and potential of h-Prune as an early tumour marker in lung cancer, a total of 14 lung cancer patients (stage I-II) (Supplementary Table S3) and 14 healthy controls were enrolled in this study and the serum levels of h-Prune were assessed using ELISA assays. As shown in figure 5b, h-Prune serum concentrations was observed to be significantly higher (*P*< 0.0005) in patients with stage I and II NSCLC than in the healthy controls (mean, 291.2±162 pg/ml and 99.3±52 pg/ml, respectively). The area under the ROC curve (AUC), as the measure of the diagnostic performance, was 0.95 (Supplemental Figure S7b). The sensitivity and specificity at a cut-off of 151,2 pg/ml were 93% and 79%, respectively.

Then, we compared the serum levels of h-Prune in additional lung cancer patients (*n* = 80, including stages I-IV) with healthy controls (*n* = 14) (Fig. 5c) (Supplementary Table S4). Serum concentrations of h-Prune ranged from 98.8 pg/ml to 980 pg/ml (mean, 294.2±191.2 pg/ml) in lung cancer patients and from 19.5 pg/ml to 183.2 pg/ml (mean, 99.3±52.3 pg/ml) in healthy controls. The area under the curve (AUC) as depicted by the ROC curve was 0.888. The sensitivity and specificity at a cut-off of 130.1 pg/ml were 83.8% and 71.4%, respectively (Supplementary Fig. S7c and d). Our results demonstrated that h-Prune levels were significantly higher in the lung cancer patients, and even more interesting in patients with early stages of NSCLC, than in the healthy controls (*P*< 0.0005; Fig. 5b-c).

We than asked whether the patients who had high serum levels of h-Prune showed also high serum levels of Wnt3a. For this reason, the serum levels of Wnt3a in lung cancer patients (*n* = 24) and healthy controls (*n* = 10) were assessed using ELISA assays. As expected, serum concentrations of Wnt3a in lung cancer patients was higher than in healthy controls (*P*= 0.03; Fig. 5d). The area under the curve (AUC) was 0.72. The sensitivity and specificity at a cut-off of 198.3 pg/ml were 95.8% and 60%, respectively (Supplementary Fig. S7e and f). Overall, the results showed that the use of Wnt3a as serum lung tumor marker had good specificity but poor sensitivity (Supplementary Table S5), in contrast, the sensitivity and the specificity of h-Prune detection alone is sufficient to identify lung cancer affected patients. In conclusion, we think that the combined use of h-Prune and Wnt3a can be of impact for lung cancer diagnosis improving sensitivity and specificity. These analyses require future efforts.

## DISCUSSION

A key observation in this study was that h-Prune is an important regulator of WNT signaling in lung cancer. The activation of the WNT pathway in lung cancer has been shown to contribute to growth and survival of lung cancer cell lines [24-27] and to promote tumor aggressiveness and resistance to chemotherapy and radiation. A recent study showed that the h-Prune C-terminal region includes an intrinsic disorder protein domain that confers the ability to bind to several different ligands [12]. Perhaps one of the most interesting features of disordered proteins is their functionality that involves binding to a partner ligand and then this interaction is associated with the induction of folding in the previously disordered structure [28]. This feature makes h-Prune an important binding partner for many proteins. Indeed, h-Prune has different binding proteins, among which, there is Gsk-3β. The h-Prune interaction domain with Gsk-3β was recently identified using NMR [3].

**Figure 6:**
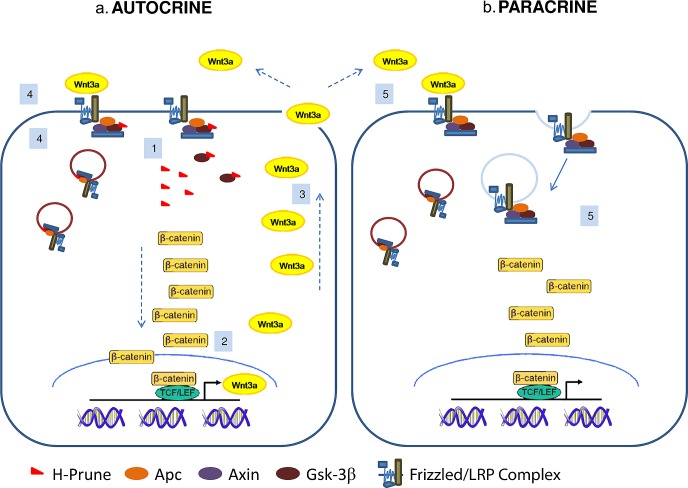
Proposed model of h-Prune action in cancer cells H-Prune binds to Gsk-3β to induce β-catenin nuclear translocation (1). As a consequence, the activation of the WNT transcriptional program promotes transcription (2) and then the release of Wnt3a (3), which has positive feedback on its own activation (autocrine) (4). Wnt3a released from the h-Prune overexpressing cells also has a paracrine effect on the other cells, leading to activation of this downstream signalling (5). These events drive h-Prune and Gsk-3β into multivesicular endosomes, further decreasing the activity of Gsk-3β in the cytosol.

Here we demonstrated how h-Prune, through its binding with Gsk-3β, takes part to the WNT/β-catenin pathway activation. Our results demonstrated that the interaction between h-Prune and Gsk-3β could potentially led to an impairment of capability of Gsk-3β to phosphorylate β-catenin. In this way, β-catenin may be not phosphorylated and becomes stabilized, accumulating in the nucleus, thus leading to activation of the WNT transcriptional programme. It is in fact consistent with our data that, as consequence of TCF/LEF activation induced by h-Prune, Wnt3a mRNA and protein expression increases, increasing also the release of the cytokine into the medium, as assessed by Elisa assay. Our data further show that Wnt3a up-regulation has as a positive consequence an enhancement of both autocrine and paracrine WNT signaling activation that drive h-Prune and Gsk-3β into multivesicular endosomes, further decreasing the activity of Gsk-3β in the cytosol, as hypothesized in the model presented in Fig. 6. Wnt signaling is regulated at different levels by a large number of effectors, either intracellularly or extracellularly, that could act as antagonists or agonists. The same β-catenin signaling leads to the activation of either positive and negative feedback pathways [14, 15]. Negative feedback mechanisms serve to restrict the duration or spread of the signaling event following the initial stimulus. For these reasons, we determined whether h-Prune activation of β-catenin signaling would also result in the activation of negative feedbacks, investigating changes in Axin2 and Dkk1 expression levels. However under our experimental conditions we did not observe increased expression levels of Axin2 and Dickkopf1 (dkk1) upon h-Prune overexpression (Supplementary Fig. S2c and d). In conclusion, we presented the evidence that Wnt3a cytokine is regulated by h-Prune, but further experiments are clearly needed to address mechanistically the early phases of this point. After characterizing the mechanism by which h-Prune induces WNT pathway activation in HEK293, cells chosen because they have an intact WNT network and are responsive to canonical ligands, including Wnt3a [8], we dissected out the function of h-Prune in lung cancer cell lines, confirming the previous results obtained in HEK293 cells. In fact, the h-Prune silencing promotes decreased proliferation and invasion in H1299 and A549 cells. We thus show that the levels of active β-catenin and the major downstream targets of the WNT pathway were severely down-regulated after h-Prune depletion, while the addition of h-Prune in these lung cancer cells lead to increase expression of active β-catenin. Together, all these findings are consistent with the involvement of h-Prune to enhance and sustain the WNT canonical pathway. Moreover, h-Prune depletion resulted in inhibition of metastases formation, which provided useful clues for the description of its mechanism of action in the invasion and metastasis formation properties of lung cells. Thus, an impairment of the expression of endogenous h-Prune results in the loss of lung cancer metastasis, and increased survival of the mice tail vein injected with NSCLC cells.

One of the first crucial clinical points in the treatment of lung cancer patients is the difficulty of obtaining an early diagnosis, which results in high mortality and poor prognosis. The patient history, physical examination, and radiological imaging studies are the gold standard for lung cancer screening. However, these tests are not sufficiently accurate for effective cancer diagnosis and disease staging [29, 30]. Serum proteins, such as carcino-embryonic antigen, cytokeratin 19 fragment, cancer antigen 125, squamous-cell carcinoma antigen, neuron-specific enolase, progastrin-releasing peptide, tumour M2-pyruvate kinase, and C-reactive protein are potential markers for lung cancer, and their levels can signify the presence of tumours, facilitate histological analysis, and allow prediction of cancer progression. However, due to their limited sensitivity and specificity, these potential markers are not recommended or encouraged in routine clinical practice. Here, we present h-Prune as a potential novel marker for the detection of early stage NSCLC, not only through its mRNA expression levels in lung cancer tissues as demonstrated by gene expression data, but also because of its secreted characteristic into peripheral blood. In diagnosis, serum cancer markers are even more important than tissue markers because they are easily procurable for large screenings for early cancer diagnosis. H-Prune has never been reported to be a secreted protein or body fluid accessible molecule. We found significantly higher levels of h-Prune expression in the serum of lung cancer patients compared to healthy controls. Early stage I or II NSCLC could even be detected with a 79% specificity and 93% sensitivity. In the near future, we will expand these analyses to investigate whether h-Prune has the potential to be developed as diagnostic marker into a population-based screening tool and can be useful for early diagnosis of NSCLC.

At this time, h-Prune would represent a selective therapeutic target for lung cancer treatment. Thus h-Prune inhibitors might prove to be pioneers for their use in preclinical studies [31] and can certainly be proposed for the prevention and treatment of h-Prune-positive lung cancer.

Wnt-3 gene expression in resected NSCLC was statistically associated with high Ki67, low apoptosis, and high expression of c-myc and survivin, and its overexpression was reported promoting tumor progression and poor prognosis in non-small cell lung cancer [32] We have demonstrated here that lung cancer patients had also high serum levels of Wnt3a. We thus believe that great importance should be given by the combination of both h-Prune and Wnt3a expression analyses in the serum in those patients with early stage of NSCLC, this approach might improve lung cancer diagnosis. This should provide helpful information for clinicians in their treatment of lung cancer patients, to reduce tumour burden and impair its metastatic disease.

In conclusion, our study not only reveals the pathological role and the regulatory mechanisms of h-Prune in NSCLC, but also indicates its correlation with tumour disease.

## MATERIALS AND METHODS

### Elisa assay

H-Prune concentrations were determined using a commercially available ELISA (Cusabio, Human ELISA kit Prune Homology-CSB-EL018831HU, China) in according to manufacturer's instructions. Wnt3a concentrations were determined using a commercially available ELISA (QAYEE, Human Wnt3a, China) in according to kit manufacturer's instructions.

### Study subjects

The study was approved by the Ethics Committee of the UOC Clinica Pneumologica SUN and the UOSD Day Hospital Oncologico Polmonare della A.O. Ospedali dei Colli, plesso Monaldi, (protocol n. 976/2013).

### Experimental lung metastasis models

To determine the effects of h-Prune silencing on the formation of metastatic lung tumours, experiments were carried out using a mouse lung metastasis model. A549_Luc cells were infected with AdV-*Sh*-UNR and AdV-*Sh*-Prune (100 MOI), and one day later they were harvested, washed, and resuspended at a density of 2 ×10^5^ viable cells/100 μL sterile PBS. The cells were injected into the tail veins of 6-week-old female nude mice. Six mice were used in each of the two groups (AdV-*Sh*-UNR and AdV-*Sh*-Prune). The mice were monitored daily and sacrificed after 11 months, following standard procedures. Mice experiments were approved by “The Institutional Animal Care and Ethical Committee of CEINGE – ‘Federico II’ University of Naples (Protocol 29, September 30, 2012; Dipartimento Sanita′ Pubblica Veterinaria D.L. 116/92).

## SUPPLEMENTARY INFORMATION FIGURES, METHODS AND TABLES


