# Supplementary material for: H-Prune through GSK-3β interaction sustains canonical WNT/β-catenin signaling enhancing cancer progression in NSCLC
Source: Oncotarget. 2014 Jul 5;5(14):5736–49. doi: 10.18632/oncotarget.2169 (PMC4170634; doi:10.18632/oncotarget.2169)
Supplement: Supplementary file 1 [file oncotarget-05-5736-s001.pdf]

H-Prune through GSK-3β interaction sustains canonical WNT/β-catenin signaling enhancing cancer progression in NSCLC

Supplementary Information

Co-immunoprecipitation assay (Supplementary Figure S1b, related to Fig. 1)

To demonstrate that Wnt pathway activation by h-Prune is exclusively due to its binding to Gsk-3β and not with other proteins, we performed co-immunoprecipitation assays. Data presented in Supplementary Fig. S1b show that h-Prune does not interact with β-catenin, Axin or Apc.

H-Prune overexpression in lung cancer cell lines enhances Wnt signaling activation (Supplementary Figure S3b-f)

Increased expression levels of active β-catenin induced by h-Prune were also observed in A549 and H1299 lung cancer cells (Supplementary Fig. S3b). Moreover, western blotting analysis showed increase expression level of Wnt3a in A549 cells overexpressing h-Prune (Supplementary Fig. S3c).

ELISA assay shown in Supplementary Fig. S3d shows that the overexpression of h-Prune in A549 cells also induces the release of Wnt3a into the medium, while there was impairment of Wnt3a expression once the h-Prune gene was silenced (Supplementary Fig. S3d-f).

Protease protection assay (Supplementary Figure S4a and b)

At the same time, in A549 cells, following h-Prune silencing through infection with adenovirus containing a shRNA against h-Prune (AdV-*Sh*-Prune), there was a significant reduction in this protection of the Gsk-3β protein in the membrane-bounded compartment with respect to control cells (Supplementary Fig. S4b).

H-Prune silencing impairs cell cycle in lung cancer cell lines (Supplementary Figure S4f and S5a)

Canonical WNT/β-catenin signaling is also involved in several biological processes, such as cell proliferation, differentiation and motility, and apoptosis. Therefore, we investigated whether the observed imbalancing pathway regulation following h-Prune expression also affects these processes. For this purpose, following h-Prune silencing, we analysed the cell cycle in lung

cancer cell lines, by monitoring the changes in the cell-cycle distribution by fluorescence-activated cell-sorting analyses (FACS) with the H1299 and A549 cell lines. As shown in Supplementary Fig. S4d, compared to the AdV-*Sh*-UNR–infected A549 cells, there was a higher fraction of cells in G1 phase at 72 h after AdV-*Sh*-Prune infection (44.07% vs. 79.65%, respectively). Similar results were obtained also in H1299 cells. These data prompted us to investigate this phenomenon further. For this reason, we evaluated the effects of h-Prune silencing on p21 and p27, as these are two important protein members of the Cip/kip family that negatively regulate G1/S phase, with inhibition of cell proliferation. As shown in Supplementary Fig. S5a, h-Prune down-regulation resulted in increased p21 and p27 protein expression that was similar in both A549 and H1299 cells, as seen using Western blotting.

Apoptosis assay (Supplementary Figure S5b)

The impairment of the cell cycle and the subsequently reduction of cell proliferation led us to also investigate the effects on caspase activation by measuring caspases 3/7 activity. As shown in Supplementary Fig. S5b, we observed that AdV-*Sh*-Prune infection in H1299 and A549 cells resulted in a significant increase in caspase activity 72 h after infection. (****P*=0.00026; *P*= **0.0013; respectively).

H-Prune silencing impairs cell migration (Supplementary Figure S5c)

We examined the effects of the knock-down of h-Prune on the cell invasion activity of A549 cells using a three-dimensional Matrigel invasion assay. As we had demonstrated that h-Prune silencing results in an increase in caspase activity at 72 h post infection (Supplementary Fig. S5b), to exclude the possibility that this effect influences cell motility, we performed these invasion assays at 48 h after infection. For this purpose, A549 lung cancer cells were infected with AdV-*Sh*-UNR and AdV-*Sh*-Prune and then seeded in 2% foetal bovine serum (FBS) medium in the upper compartment of the invasion plates. Invasion was quantified by counting the stained cells adherent to the lower side of the membranes for each of three chambers for each condition. A mean of 203 (±19.3) AdV-*Sh*-UNR–infected cells per high-power field had migrated onto the filter surface, whereas only 24.6 (±7.2) AdV-*Sh*-Prune–infected cells reached the filter (*P*= 0.00011) (Supplementary Fig. S5c). Thus, these reduced numbers of AdV-*Sh*-

Prune–infected cells on the filter surface indicated that down-regulation of h-Prune expression mitigates the migration of these A549 cells *in vitro*.

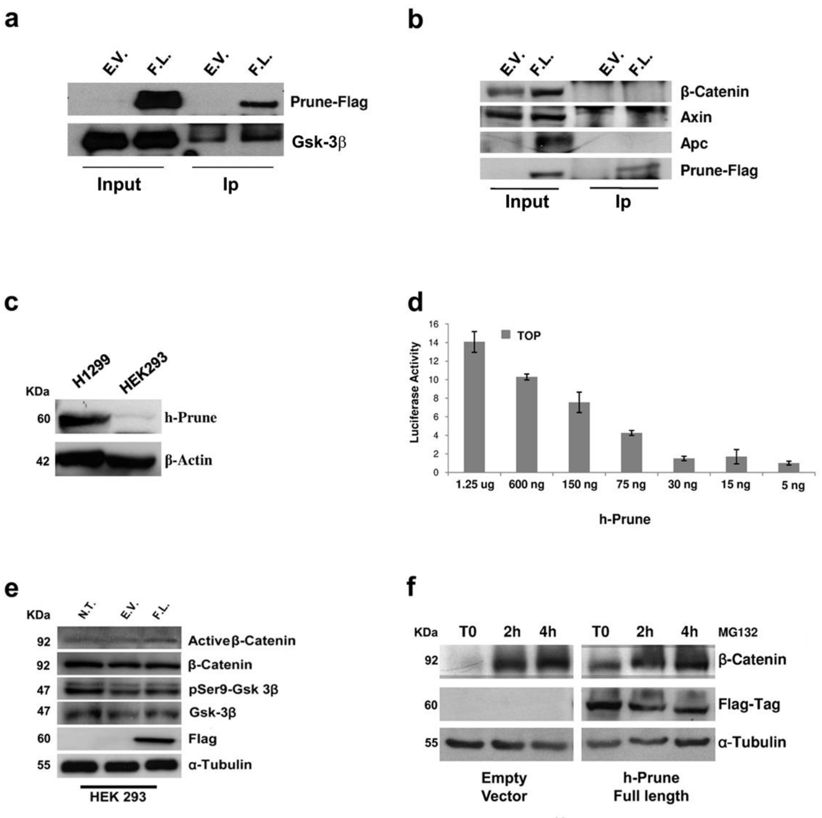

Supplementary Figure 1:(a) GSK-3β and h-Prune interact in A549 cells. H-Prune cDNA was single transfected in A549 cells. H-Prune retains the FLAG tag. Total protein extract from empty vector (E.V.) and full-length h-Prune (F.L.) was incubated overnight with the anti-GSK-3β antibody to immunoprecipitate endogenous GSK-3β. The band of expected size after Western blotting using an anti Flag antibody reveals the presence of h-Prune bound to GSK-3β (upper panel, right). **(b)** Co-immunoprecipitation showing that h-Prune does not interact with β-catenin, Axin or Apc. The anti-FLAG antibody was used to immunoprecipitate and detect h-Prune. **(c)** Endogenous levels of h-Prune in H1299 and HEK293 cells. β-Actin was used as loading control. **(d)** H-Prune induces β-catenin-mediated transcription activity in a dose-dependent manner. Cells were transfected with TOP/FOP flash plasmid and Renilla luciferase plasmid overnight. Values were normalized to Renilla luciferase activity. **(e)** Western blotting showing increased expression levels of active β-catenin in h-Prune overexpressing cells. Total β-catenin remains unchanged. □-Tubulin was used as the loading control and the anti-Flag antibody provides the transfection control. **(f)** Total proteins prepared from HEK293 cells transfected with control vector or full-length h-Prune, which were exposed to MG132 for different times (0h, 2h, 4h), were subjected to Western blotting with anti-β-catenin. α-tubulin was used as the loading control and the anti-Flag antibody provides the transfection control.

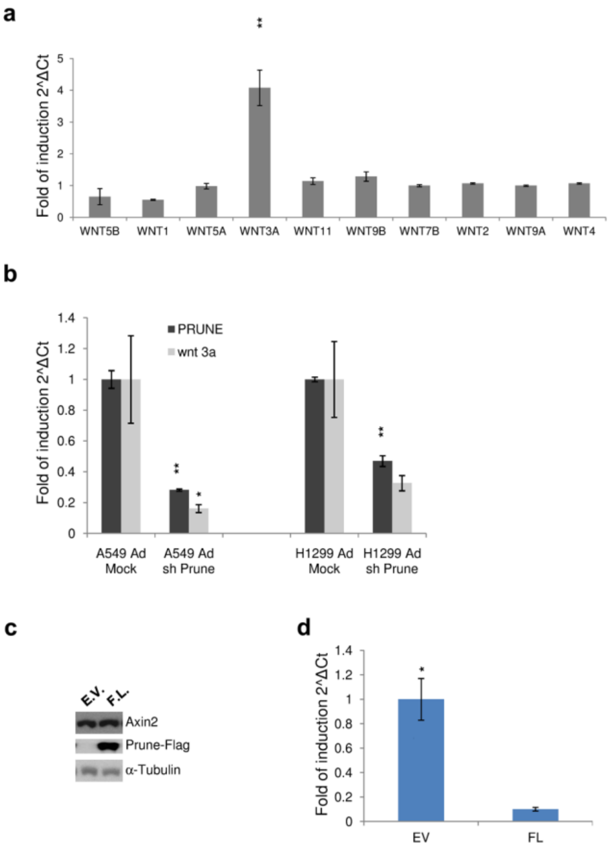

Supplementary Figure 2 :(a) mRNA expression levels of Wnt cytokines in Hek293 cells transfected with the empty vector and the full-length h-Prune vectors, as determined by qRT-PCR. The levels of mRNA expression are represented as fold-multiples of 2-DCT values relative to empty vector expression (***P* = 0.007). **(b)** mRNA expression levels of Wnt3a in AdV-*Sh*-UNR- and AdV-*Sh*-Prune-infected A549 cells, as determined by qRT-PCR. The levels of mRNA expression are represented as fold-multiples of 2-DCT values relative to empty vector expression. Data are means (mRNA expression 2-DCt) ±SD (n = 3)(**P* = 0.05; ***P* < 0.005). **(c)** Western blotting analysis to compare Axin2 expression in Hek293 cells transfected with the empty vector and the full-length h-Prune vectors. α-Tubulin was used as the loading control and the anti-Flag antibody provides the transfection control. **(d)** mRNA expression levels of Dkk1 in Hek293 cells transfected with the empty vector and the full-length h-Prune vectors, as determined by qRT-PCR. The levels of mRNA expression are represented as fold-multiples of 2-DCT values relative to empty vector expression. Data are means (mRNA expression 2-DCt) ±SD (n = 3) (**P* = 0.017).

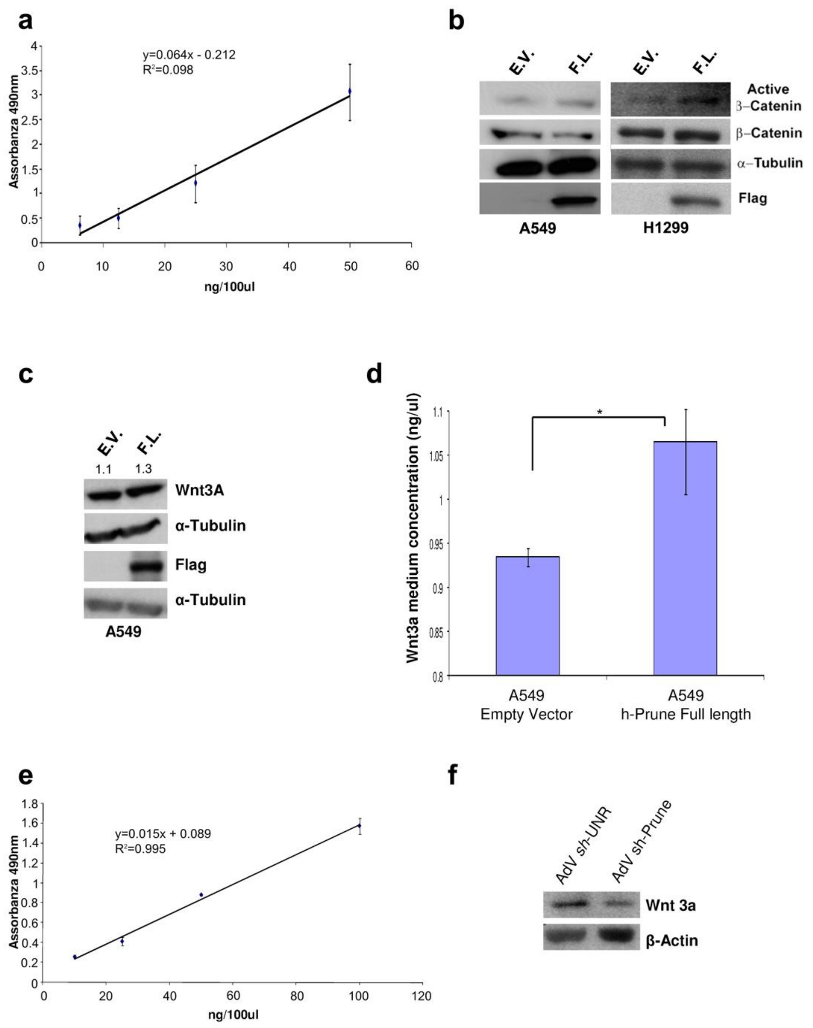

Supplementary Figure 3 :(a) Standard curve for the direct ELISA in Fig. 2c, with rhWnt3a protein at a range of 50 ng to 6.25 ng/100µl. Data are means ±SD (n = 2). **(b)** Western blot showing that the overexpression of full-length h-Prune in A549 and H1299 cells leads to increased active β-catenin expression, compared to empty vector transfected cells. The β-catenin levels remained unchanged. The efficiency of transfection was assessed using an anti-Flag antibody. α-Tubulin was used as the loading control. **(c)** Western blot showing increased amounts of Wnt3a in h-Prune-overexpressing A549 cells. The efficiency of transfection was assessed using an anti-Flag antibody. α-Tubulin was used as the loading control. **(d)** ELISA for Wnt3a levels in conditioned medium from A549 cells overexpressing h-Prune, as compared to the empty vector (*P = 0.02). **(e)** Standard curve for the direct ELISA in Fig. S3d,with rhWnt3a protein at a range of 100ng/100µl to 10 ng/100µl. Data are means ±SD (n = 2). **(f)** Western blot showing that expression of Wnt3a was reduced in AdV-*Sh*-Prune- compared to AdV-*Sh*-UNR-infected A549 cells.

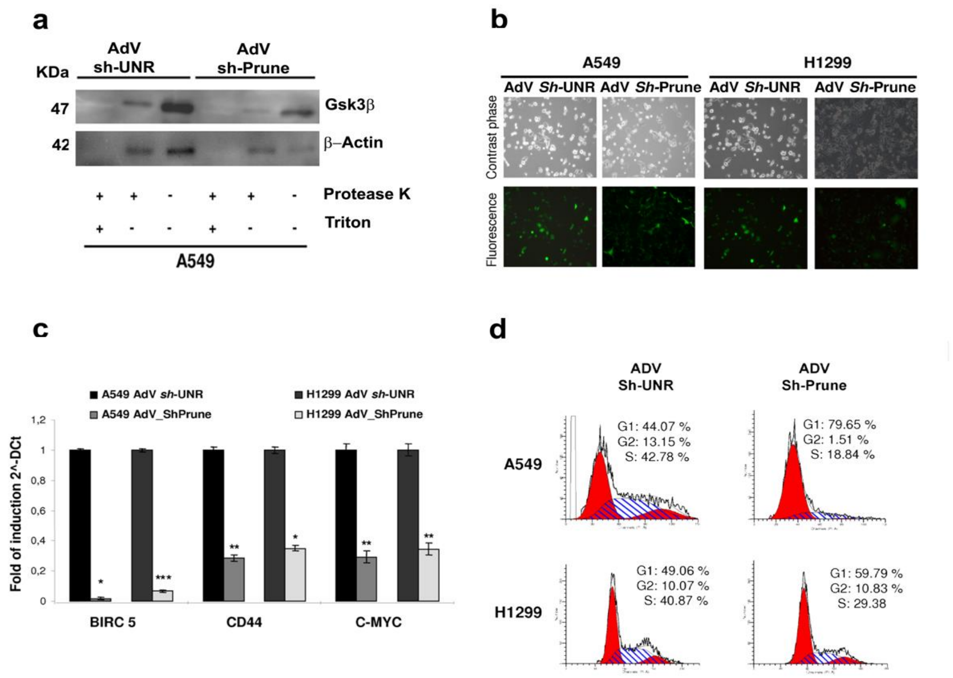

Supplementary Figure 4 :(a) Western blots of protease protection assay in AdV-*Sh*-UNR-and AdV-*Sh*-Prune-infected A549 cells. Cells were digitonin-permeabilized, and membranes were isolated and incubated without or with proteinase K in the absence or presence of Triton X-100. Gsk-3β wasn't protected from Proteinase K in AdV-*Sh*-Prune-infected A549 cells. **(b)** Representative images showing efficiency of H1299 and A549 cell lines infection with adenovirus carrying GFP. Upper panels: Phase contrast microscopy. Lower panels: Fluorescence. **(c)** mRNA expression levels of BIRC5, CD44 and C-MYC in AdV-*Sh*-UNR- and AdV-*Sh*-Prune-infected A549 and H1299 cells determined by qRT-PCR. Data are means ±SD (n = 2)(*$P < 0.05$; **$P < 0.005$; ***$P < 0.0005$). **(d)** H-Prune silencing in A549 cells by adenoviral infection (AdV-*Sh*-Prune) induces increased A549 and H1299 cell arrest in G1 phase (79.65% and 59.79%, respectively), compared to the control cells (44.07% and 49.06%, respectively), as determined by PI-based FACS analysis.

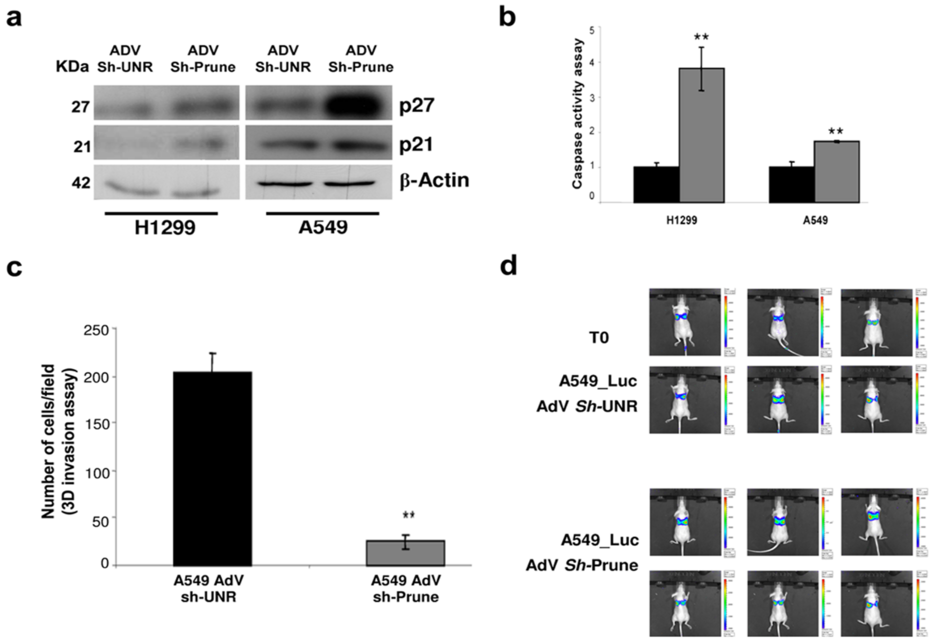

Supplementary Figure 5:(a) Western blotting detection of increased expression of p21 and p27 after h-Prune silencing in H1299 and A549 cells. β-Actin was used as the loading controls. **(b)** Analysis of caspase-3 activity in H1299 and A549 lung cancer cell lines infected with AdV-*Sh*-UNR and AdV-*Sh*-Prune. H-Prune reduction by adenoviral infection (AdV-*Sh*-Prune) induces significant increases in caspase-3 activity, as monitored by fluorimetric caspase assay, in both H1299 cells (****P* = 0.00026) and A549 cells (***P*= 0.0013), as compared to the control vector (AdV-*Sh*-UNR)-infected cells. (**c**) The knock-down of h-Prune expression reduced A549 cell invasion *in vitro*. Cells invading the matrigel were fixed and stained with hematoxylin-eosin. The numbers of invading cells were counted under a microscope. Data are means ±SD (n = 3). Statistical analysis was performed by Student's t-tests. ***P*<0.01, compared with A549 AdV-*Sh*-UNR infected cells. **(d)** Two NOD/SCID mice groups following tail-vein injections of A549-Luc cells previously infected with AdV-*Sh*-UNR (6 mice) and AdV-*Sh*-Prune (6 mice). The injections (at T0) were followed by *in-vivo* bioluminescence photon emission signals (IVIS Imaging System) (Related to Fig. 4c).

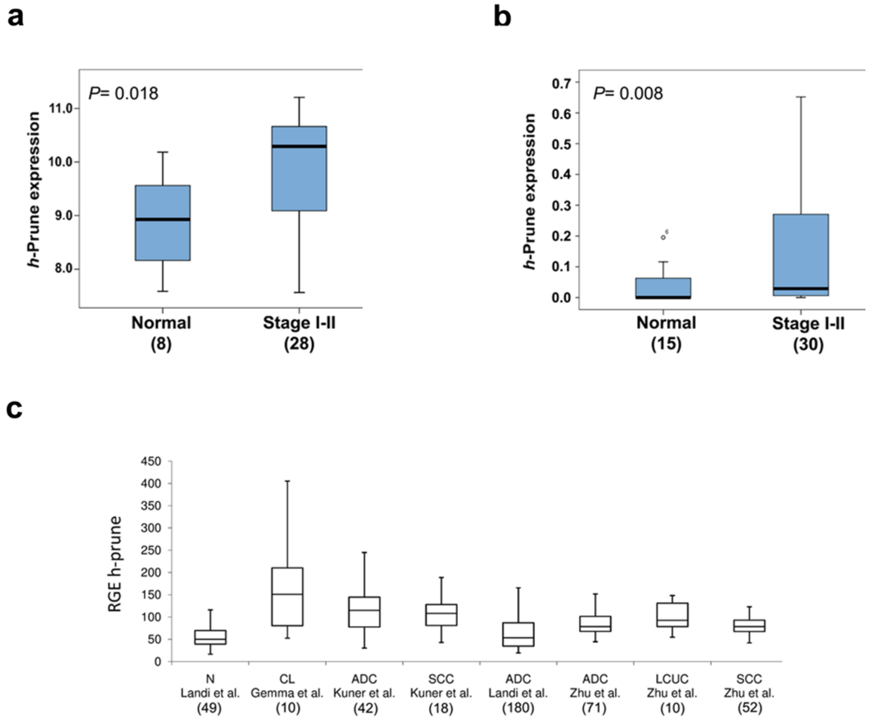

Supplementary Figure 6: (a) Expression of h-Prune determined by gene expression microarray. H-Prune expression was assessed in mRNA NSCLC tissues (n= 28) and compared to adjacent non tumour tissues (n=8), with the results represented as boxplots. Differential expression was evaluated between stages 1-2 and compared to normal lung tissues (Normal). H-Prune expression is significantly higher in patients at stage I-II (*P* = 0.018), when compared to non tumour tissues (Normal). **(b)** H-Prune expression was assessed by qRT-PCR in NSCLC tissues (n=30) and compared to adjacent non tumour tissues (n=15), with the results represented as boxplots. H-Prune expression is significantly higher in patients at stage I (*P*=0.008), if compared to non tumour tissues. **(c)** Relative gene expression of h-Prune in 4 lung cancer datasets: RPLP0 was used to normalize the expression of h-Prune, in each dataset, as median exp RPLP0 x (exp h-Prune / exp RPLP0) with exp corresponding to the median of the probset signal values. N: normal lung (Landi et al.), CL: Cell lines (Gemma et al.), ADC : Adenocarcinoma (Kuner et al., Landi et al. And Zhu et al.), SCC: Squamous cell carcinomas (Kuner et al. And Zhu et al.), LCUC: Large Cell Undifferentiated Carcinoma (Zhu et al.).

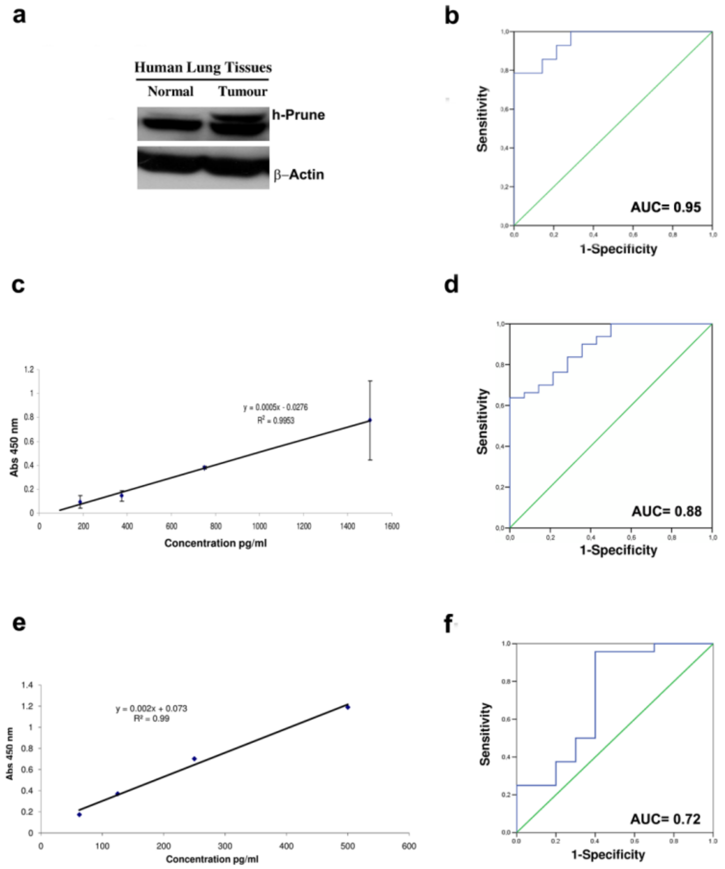

Supplementary Figure 7: (a) Western blot showing h-Prune expression in human lung cancer tissue, compared to normal lung tissue. β-Actin was used as the loading control. **(b)** ROC curve for ELISA (related to Fig. 6b). The area under the curve (AUC) was 0.95. The sensitivity and specificity at a cut-off of 151.2 pg/ml were 93% and 79%, respectively. **(c)** Standard curve of the recombinant h-Prune protein for the sandwich ELISA, for the range of 1500 pg/ml to 94 pg/ml. Data are means ±SD (n = 2). **(d)** ROC curve for ELISA (related to Fig. 6c). The area under the curve (AUC) was 0.888. The sensitivity and specificity at a cut-off of 130.1 pg/ml were 83.8% and 71.4%, respectively. **(e)** Standard curve of the recombinant Wnt3a protein for the sandwich ELISA, for the range of 500 pg/ml to 62.5 pg/ml. Data are means ±SD (n = 2). **(f)** ROC curve for ELISA (related to Fig. 6d). The area under the curve (AUC) was 0.725. The sensitivity and specificity at a cut-off of 198.3 pg/ml were 95.8% and 60%, respectively.

Methods

Tissue samples

Lung tumour tissue specimens and adjacent non-cancerous tissues were obtained sequentially from NSCLC patients surgically resected at the Monaldi Hospital, Naples, Italy from 2004 to 2007. All of the tissue samples were collected at the time of surgery, immediately flash frozen in liquid nitrogen, and stored at -80°C until use. The histologies of tumour types and the postoperative stages were determined according to the WHO classification method [1] and the Tumor-Node-Metastasis system [2], respectively.

RNA extraction and quantitative real-time PCR

Total RNA was extracted from tissues or cells using Trizol reagent (Invitrogen) according to the manufacturer recommendations, and were treated with RNase free DNase (Promega). To facilitate RNA extraction from tissues, the samples were first homogenised in TRIzol Reagent using a TissueLyser (Qiagen, Valencia, CA, USA).

Total RNA (1 μg) was reverse transcribed using iScript™ cDNA Syntesis kits (Biorad). The reverse transcriptase products (cDNA) were amplified by real-time quantitative PCR using a 7900 Real-Time PCR System (Applied Biosystems, Foster City, CA, USA) with Power SYBR green Master Mix. For all of the Syber Green PCR, Ct values were normalized to β-actin. Relative expressions of target genes were determined by the $2^{-\Delta\Delta Ct}$ method. All of the data are presented as means ±standard error of two to three replicate experiments.

Luciferase report assay

For the luciferase assay, HEK293T cells were plated into 96-well plates at a density of 10^5 cells/well, and incubated overnight at 37°C.

The cells were transfected using the calcium phosphate method, with 25ng of each TOP-FLASH reporter plasmid (gift from H. Clevers, Hubrecht Institute, Royal Netherlands Academy of Arts and Sciences, 3584 CT Utrecht, the Netherlands) containing 4 Tcf consensus binding sites upstream of firefly luciferase cDNA, or a FOP-FLASH reporter plasmid as negative control, containing a mutated Tcf binding site [3].

Cells were also transfected with 65 ng of each h-Prune full-length flag-tagged plasmid and with the empty control vector, and incubated at 37°C. After 24 h, the medium was replaced with fresh

serum-free medium and the cells were starved by serum deprivation overnight. Drug treatments were given for 3 h (IGF-1, Chiron-99021, SB-216763).

The reporter assay was performed using the Dual-Luciferase® Assay reporter system (Promega), according to the manufacturer instructions. The firefly and renilla luciferase activity were measured using a luminometer (Perkin Elmer, EnVision 2102 Multilabel Reader).

Immunoflourescence assay

Immunofluorescence staining was carried out with HEK293cells cultured on cover slips. The cells were fixed in 4% paraformaldehyde and permeabilised for 10 min in phosphate buffer containing 0.1% Triton X-100. The relevant antibodies were then added to a blocking buffer (0.1% Triton X-100, 10% pig serum) at the dilutions recommended by the manufacturers. Confocal microscopy was carried out using a laser scanning confocal microscope LSM 510 META, Zeiss, with 40x/63x water immersion objectives.

Western blotting

Tissues were homogenised with a TissueLyser [Qiagen], according to the manufacturer instructions. Cells were washed in cold phosphate-buffered saline and lysed in protein lysis buffer (20mmol/L sodium phosphate, pH 7.4, 150mmol/L NaCl, 10% glycerol, 1% Na-deoxycholate and 1% Triton-X), supplemented with protease inhibitors (Roche).

Protein concentrations were determined using a Bradford assay kit (Bio-Rad) with bovine serum albumin as the standard. The protein (50 μg) was run on SDS-PAGE gels and transferred to PVDF membranes (Millipore). The membranes were blocked for 1h at room temperature and incubated with the primary antibody overnight at 4 °C, and with the secondary antibody for 1 h at room temperature. The signal was visualised using an enhanced chemiluminescence detection system (ECL Plus, Pierce), according to the manufacturer instructions.

The antibodies used were as follows: anti-Gsk-3β (BD Biosciences 610201; dilution 1:500); anti-phospho-GSK-3α/β (Ser21/9; Cell Signaling #9331; dilution 1:500); anti-active-β-catenin (Millipore clone 8E7; dilution 1:500); anti-cyclinD1 (A-12; Santa Cruz 8396; dilution 1:200); anti-survivin (Ab 469; dilution 1:1000); anti-c-Myc (9E11) sc-47694.

Cell culture and infection

A549 and H1299 cells were grown under standard conditions in DMEM and RPMI, respectively, containing 10% (v/v) foetal calf serum, 2 mM L-glutamine, and 1% pen/strep. Infection with recombinant viruses was accomplished by exposing the cells (100 MOI/cell) to adenovirus in 500 µl complete cell culture medium for 1 h, followed by addition of other media.

Immunohistochemistry

Paraffin sections (5-6-µm thick) were deparaffinised and placed in a solution of absolute methanol and 0.3% hydrogen peroxide for 30 min. They were then washed in PBS before immunoperoxidase staining. The slides were then incubated overnight at 4°C in a humidified chamber with the anti-h-Prune polyclonal antibody diluted 1:100 in PBS and an anti-active β-catenin mouse monoclonal antibody (Millipore) diluted 1:75 in PBS. Tissue sections were then incubated, first with biotinylated goat anti-rabbit and anti-mouse IgG for 20min (Dako Real ™ Detection System, Dako) and then with streptavidin conjugated to horseradish peroxidise reagent (Dako) for 20 min. The immunostaining was performed by incubating the slides in diaminobenzidine (Dako) solution containing 0.06 mM diaminobenzidine and 2 mM hydrogen peroxide in 0.05% PBS (pH 7.6) for 5 min. After chromogen development, the slides were washed, dehydrated with alcohol andxylene, and mounted with cover slips using a permanent mounting medium (Permount). Micrographs were taken with a Leica DFC320 digital camera.

Soft agar assay

Anchorage-independent growth of A549 and H1299 cells was examined in semisolid agar. This assay was made preparing a solution of 2% agar (DNA grade), melted in microwave, and 2× DMEM/RPMI (A549/H1299 cells, respectively) with 10%FBS, 2.0 mM L-glutamine, 100 U/ml pen-strep in an equal volume, with 2 ml of this agar solution added to each well of six-well tissue culture plates and stored at 4 °C for 2 h. A volume of 2 ml of 0.35% agar and DMEM/RPMI 2× solution containing 1.5×10^5 cells infected with AdV-*Sh*-UNR or AdV-*Sh*-Prune was then layered over the bottom agar in each well, in duplicate. The plates were incubated in 5% CO_2 at 37°C in the presence of 10% FBS/DMEM for 4 weeks. The cells were stained with crystal violet 0.005% and assayed based on different sizes with the aid of a Leica microscope. The significant differences between the three different treatments were assessed using T-tests.

Matrigel assay

The impact of down-regulation of h-Prune expression on the invasiveness of A549 cells was determined using Transwell chambers (Chemicon International Inc., Temecula, CA, USA) according to the manufacturer instructions. Briefly, the cells were infected with the h-Prune-specific shRNA adenovirus or the control adenovirus. Two days after infection, the cells were seeded into the upper compartment of Matrigel coated plates at a concentration of 40,000 cells in 500 µL 2% FBS medium. The lower compartment was filled with 6% FBS medium and incubated at 37°C for 12h. The cells were fixed to the bottom of the chamber with 95% ethanol/ 5% acetic acid, and stained with hematoxylin-eosin. Invasion was quantified by counting the stained cells adherent to the lower side of the membranes, for each of three chambers for each condition.

Cell treatments with proteasome (MG132) and lysosome acidification (NH_4Cl) inhibitors

HEK293 cells were transfected with pCS4-3xFlag empty vector and pCS4-3xFlag-Prune. After 36h, transfected cells were treated with 20 µM MG132 (Sigma, cat. C2211) or 30 mM NH_4Cl, and harvested at different times, for the total protein extract. As control, we used protein extracts from untreated cells loaded on SDS PAGE together with the treated samples.

Cell proliferation assay by xCELLigence

A549 and H1299 cells were seeded in 16-well plates (E-plate 16, Roche, Mannheim, Germany) (2×10^5 cells in 100 µl medium/well), following the xCELLigence Real-Time Cell Analyzer DP instrument manual, as provided by the manufacturer (Roche). After 2 h, AdV-*Sh*-UNR or AdV-*Sh*-Prune (100 MOI) was added, and the experiment was allowed to run for 72 h. Average baseline cell index and percentage reduction of the baseline cell index for infected cells compared to control cells were calculated for at least two measurements from 4 replicate experiments, ±SD. Significance was calculated using one-tailed Student's t-tests ($P < 0.05$).

Measurement of Wnt3a expression levels in culture media

The levels of the Wnt3a cytochine were measured using ELISA. After transfection of the empty vector or h-Prune, 500 µl medium was drawn from the culture plates 36 h later. After

centrifugation at 2000 rpm for 10 min, all of the samples were assayed in duplicate using BD Falcon Microtest96-well ELISA Plate Clear, with prior equilibration with carbonate/bicarbonate buffer, pH 9.3. Then 100 μl of each sample and the rhWnt3a protein (R&D Systems, Inc.) that had been diluted with carbonate/bicarbonate buffer, pH 9.3,to the range of 100 ng to 0.001ng, were added in triplicate and the plates were incubated overnight at 4°C. The next day, the plate was washed with PBS with 0.1% Tween-20 (Carlo Erba), and blocked with 1% bovine serum albumin for 1 h at room temperature. Then, after two washes with PBS and 0.1% Tween-20, the Wnt3a detection antibody (ab28472) diluted 1:200 in PBS and 0.1% Tween-20 was added for 1 h at room temperature. After three washes with PBS with 0.1% Tween-20, the goat anti-rabbit IgG (H and L) lyophilized HRP (ImmunoReagents Inc.) was diluited 1:200 with PBS with 0.1% Tween-20, added, and incubated for 1 h at room temperature. Finally, after 3 washes with PBS with 0.1% Tween-20, 100 μl of the o-phenylenediamine color reagent (Sigma) dissolved in citrate buffer, pH5, and activated with 10 μl H_2O_2 per ml solution, was added to the plate. The reaction was allowed to develop in the dark for 15 min and was then interrupted by the addition of 30% H_2SO_4 to each well. The absorbance was read on a plate reader at 490nm (Elisa Microplate-Biorad).

Protein protection assay

Protease protection assays were performed essentially as described by Vanlandingham and Ceresa (2009) [4]. Briefly, untransfected HEK293 cells, pCS4-3xFlag empty vector cells, and pCS4-3xFlag-Prune transfected cells were harvest 24 h after transfection, and mechanically detached from a 10 cm culture plate by pipetting with PBS. The cells were pelleted at 800 rpm and then resuspended in RB Buffer (100 mM potassium phosphate, pH 6.7, 5 mM $MgCl_2$, 250 mM sucrose) containing 6.5 mg/ml digitonin, and incubated for 5 min at room temperature, followed by 30 min on ice. The cytoplasmic fraction was obtained by removing the nuclei by centrifugation for 10 min at 800 rpm. The supernatant was transferred into clean tubes and the cellular bodies were obtained by centrifugation for 10 min at 13,000 rpm. Then these bodies were resuspended in 3 aliquots and mixed with an equal volume of buffer with: 1 mg/ml proteinase K (Invitrogen), 1 mg/ml proteinase K and 0.1% Triton X-100, or resuspension buffer. The cells were incubated for 15 min at room temperature and the proteinase K digestion was

stopped by adding 20 mM PMSF in 4× loading buffer, and heated to 95°C for 10 min before electrophoresis.

Cell-cycle analysis

Cells were infected cells with AdV-*Sh*-UNR and AdV-*Sh*-Prune at 100 MOI. Then 72 h after infection, thecells were harvested by brief trypsinization and were washed twice with ice-cold PBS, with the cell pellets collected. Approximately 0.5×10^5cells were fixed in 96% ethanol for 4 h, and then were treated with 50 U/ml RNase-A and labelled with 20 µg/ml propidium iodide and were incubated at 4°C overnight in the dark. The percentage of cells in each phase of the cell cycle was determined by fluorescence-activated cell sorting (FACS) (Becton Dickison, Franklin Lakes, NJ, USA) and analyzed using the ModFit LT 2.0 1995-6 software (Verity Software House Inc.).

Caspase-3 activity assay

A549 and H1299 cells were infected with AdV-*Sh*-UNR and AdV-*Sh*-Prune, as previously described, and caspase-3 activity assays were performed. After 72 h, the cells were harvested and rinsed in PBS, and then lysed in RIPA buffer, for total cell lysates. Protein concentrations were determined using Bradford assay kits (Bio-Rad) with bovine serum albumin as the standard. The caspase-3 activity assay was performed using BD Pharminge Ac-DEVD-AFC Caspase-3 Fluorogenic Substrate, according to the manufacturer instructions. The absorbance for each well was measured at 480 nm using a Victor3 Multilabel counter (Perkin Elmer). Each experimental point was performed as four replicates, and each experiment was repeated in duplicate.

Gene expression data mining

Microarray analysis was performed in triplicate, and each replicate used normal and tumor lung tissues. We used an array platform that was provided by Illumina and hybridized RNA extracted from 50 lung cancer samples, and also from respective normal tissue using DASL Assays (**Illumina® Gene Expression Profiling)** and hybridization, washing, staining, and scanning of the chip was performed according to the manufacturer instructions. Our analysis was saved into the default folder inside the ANALYSIS directory. Before proceeding to the next step, we transformed the expression values to log scale (log2) for all of the subsequent analysis on the

log-transformed values. Taking advantage of the array QualityMetrics package we created a quality assessment report that incorporated a set of assessment plots, including sample quality assessment and quality diagnostics. All of the quality plots were saved in the a QM directory. As non biological factors can greatly contribute to the variability of data from different arrays we normalized across the chips, to reduce this source of unwanted variation. We used the facilities exposed in lumi and beadarray to background correct, normalize and summarize the probe level data into expression level data, as described in [5]. Then, an Expression Set object was built up that contained the expression level data, the target information, and both the sample and feature annotations.

Missing values frequently pose problems in gene expression microarray experiments, as they can hinder the downstream analysis of the datasets. According to [6], missing value imputation always gave better results than ignoring missing data points or replacing them with zeros or average values. So we decided to impute the normalized data expression matrix, taking advantage of the impute package. After these preliminary steps, we selected the genes that were differentially expressed across the contrast of interest, taking advantage of the moderated t-test analysis included in the limma [7] package.

Statistical analyses

We compared h-Prune expression in each lung tumour sample at different histology grades to that of non-cancer samples. All of the data are presented as means ±standard error. Statistical significance was calculated using the Mann-Whitney test. The difference was considered statistically significant at $P<0.05$. All statistical analyses were performed using the SPSS 16 statistical package for Windows.

References

1. Brambilla E, Travis WD, Colby TV, Corrin B and Shimosato Y. The new World Health Organization classification of lung tumours. Eur Respir J. 2001; 18(6):1059-1068.

2. Wittekind C, Compton CC, Greene FL and Sobin LH. TNM residual tumor classification revisited. Cancer. 2002; 94(9):2511-2516.

3. Lee JS, Ishimoto A and Yanagawa S. Characterization of mouse dishevelled (Dvl) proteins in Wnt/Wingless signaling pathway. The Journal of biological chemistry. 1999; 274(30):21464-21470.
4. Vanlandingham PA and Ceresa BP. Rab7 regulates late endocytic trafficking downstream of multivesicular body biogenesis and cargo sequestration. The Journal of biological chemistry. 2009; 284(18):12110-12124.
5. Du P, Kibbe WA and Lin SM. lumi: a pipeline for processing Illumina microarray. Bioinformatics. 2008; 24(13):1547-1548.
6. Tuikkala J, Elo LL, Nevalainen OS and Aittokallio T. Missing value imputation improves clustering and interpretation of gene expression microarray data. BMC bioinformatics. 2008; 9:202.
7. Smyth GK, Michaud J and Scott HS. Use of within-array replicate spots for assessing differential expression in microarray experiments. Bioinformatics. 2005; 21(9):2067-2075.

Supplementary Table 1: (related to Supplementary Fig. S6a and b). Summary of the cases analyzed using the microarray platform and RT-PCR.

Stage	Microarray platform			Real time		
	N	**%**	*P*	**N**	**%**	*P*
I-II	28	78	*0.018*	30	67	0.008
Normal tissues	8	22		15	33	
**Total**	**36**	**100**		**45**	**100**	

Supplementary Table 2: (related to Fig. 5a). Immunohistochemical analysis of h-Prune and active β-catenin protein expression in normal and neoplastic lung tissues.

Staining scores: 0, no positive cells; 1+, <10% positive cells; 2+, 11%-60% positive cells; 3+, 61%-100% positive cells.

Tumor histotype	No. of cases	H-Prune				Active β-Catenin			
		Neg	Positive			Neg	Positive		
		0	1+	2+	3+		1+	2+	3+
Normal lung tissue	3	2	1			1	2		
Squamous cell	11		1	3	7		1	5	5
Adenocarcinoma	13		1	4	8	2	1	5	5
Large cell	6		1	2	3	1	2	2	1

Supplementary Table S3: Characteristics of patients with stage I-II NSCLC enrolled in this study (related to ELISA assays presented in Fig. 5b).

Patient ID	H-Prune pg/ml	Age	Hystology	T	N	M	Stage
B103	164.3	72	Adenocarcinoma	T1b	N0	M0	IA
B143	206.8	65	Adenocarcinoma	T1b	N0	M0	IA
B206	337.6	61	Adenocarcinoma	T1b	N0	M0	IA
B224	749.3	45	Adenocarcinoma	T1b	N0	M0	IA
B224	410.3	45	Adenocarcinoma	T1b	N0	M0	IA
B25	328.2	53	Adenocarcinoma	T1a	N0	M0	IA
B100	443.2	56	Adenocarcinoma	T2a	N0	M0	Ib
B116	261	67	Adenocarcinoma	T2	N0	M0	IIA
B129	137.6	58	Adenocarcinoma	T1b	N1	Mx	IIA
B135	287.6	67	Adenocarcinoma	T2b	N0	Mx	IIA
B191	191	76	Adenocarcinoma	T2a	N1	M0	IIA
B63	157.2	71	Adenocarcinoma	T2a	N0	M0	IIA
B12	205.8	73	Adenocarcinoma	T4	N0	M0	IIB
B349	196.6	73	Adenocarcinoma	T3	N0	Mx	IIB

Supplementary Table S4a: Characteristics of patients enrolled in this study (related to ELISA assays presented in Fig. 5c and d).

Characteristic	H-Prune		Wnt3a	
	Patients (n=80)	(pg/ml)	Patients (n=24)	(pg/ml)
**Median age (years)**	63±9	294.2±191.2	65±9	284.4±135.8
**Gender**				
**Female**	22		8	
**Male**	58		16	

Supplementary Table S4b*:* Characteristics of healthy controls enrolled in this study (related to ELISA assays presented in Fig. 5c and d).

Characteristic	H-Prune		Wnt3a	
	Healthy controls (n=14)	(pg/ml)	Healthy controls (n=10)	(pg/ml)
**Median age (years)**	59±15	99.3±52.3	58±12	200.9±55
**Gender**				
**Female**	4		3	
**Male**	10		7	

Supplementary Table S5: Use of normal cut-off values of serum tumor markers for lung cancer diagnosis

	**PRUNE**	**WNT3A**
AUC (95% CI)	0.888	0.725
Positive boundary value	>130.1 pg/ml	> 198.3 pg/ml
Sensitivity (%)	83.8	95.8
Specificity (%)	71.4	60
